# Supplementary material for: Promotion of Lymphangiogenesis by Targeted Delivery of VEGF-C Improves Diabetic Wound Healing
Source: Cells. 2023 Feb 1;12(3):472. doi: 10.3390/cells12030472 (PMC9913977; doi:10.3390/cells12030472)
Supplement: Supplementary file 1 [file cells-12-00472-s001.zip › cells-2174427-supplementary.pdf]

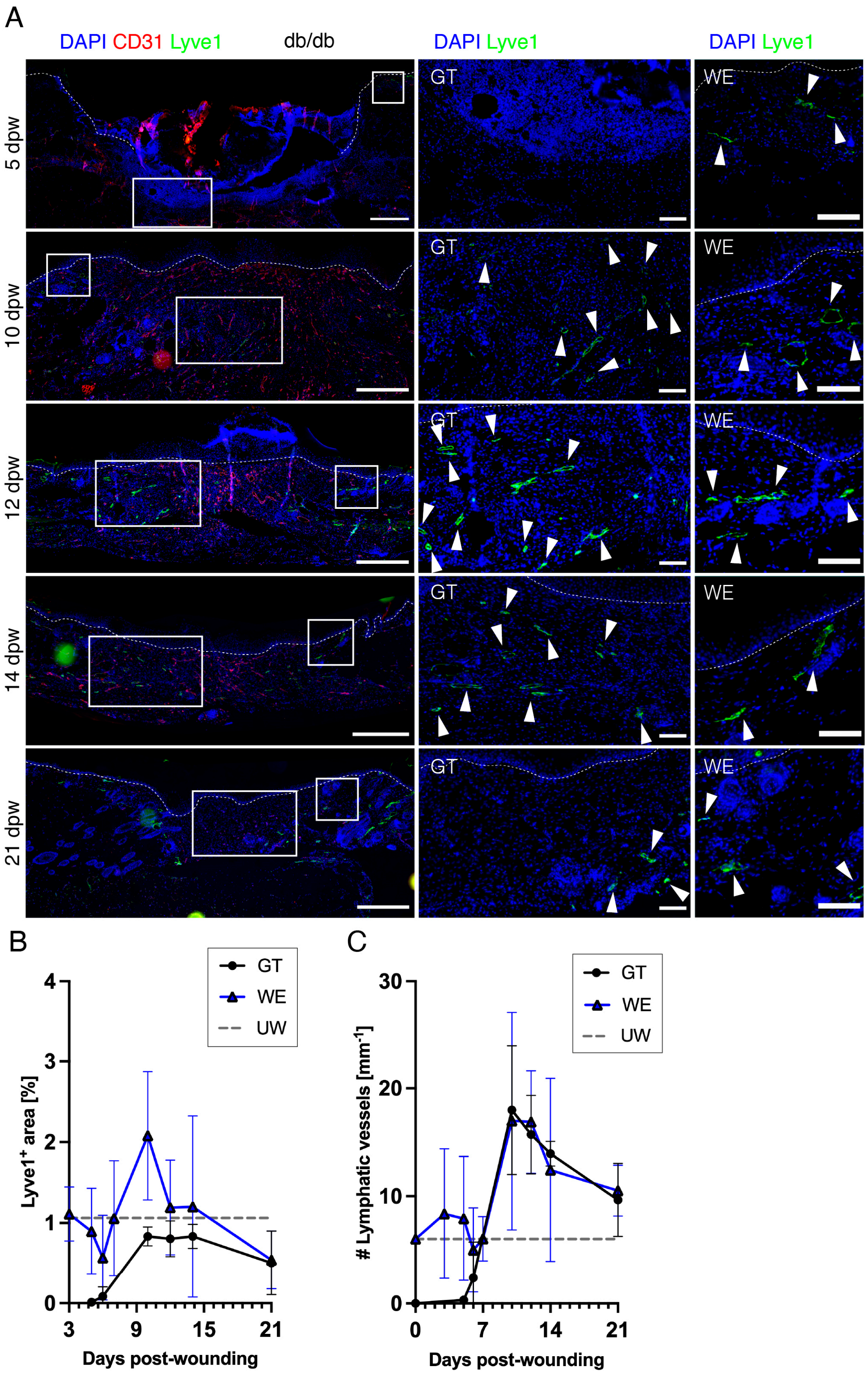

Figure S1. Wound lymphangiogenesis in diabetic mice peaks around 10 days post-wounding. (A) LYVE1-CD31 representative sections of diabetic wounds at 5-, 10-, 12- and 14- and 21-days post-wounding (dpw). Left to right: Overview, granulation tissue (GT), wound edge (WE). Scale bars: Overview 500 µm, GT, WE: 200 µm. **(B)** Quantification of the LYVE1^{+} area [%] of LVs in representative regions of wound sections. **(C)** Quantification of the number of LVs [mm^{-1}] in representative regions of wound sections. N = 2-6 wounds per day of 11 mice. UW: unwounded skin.

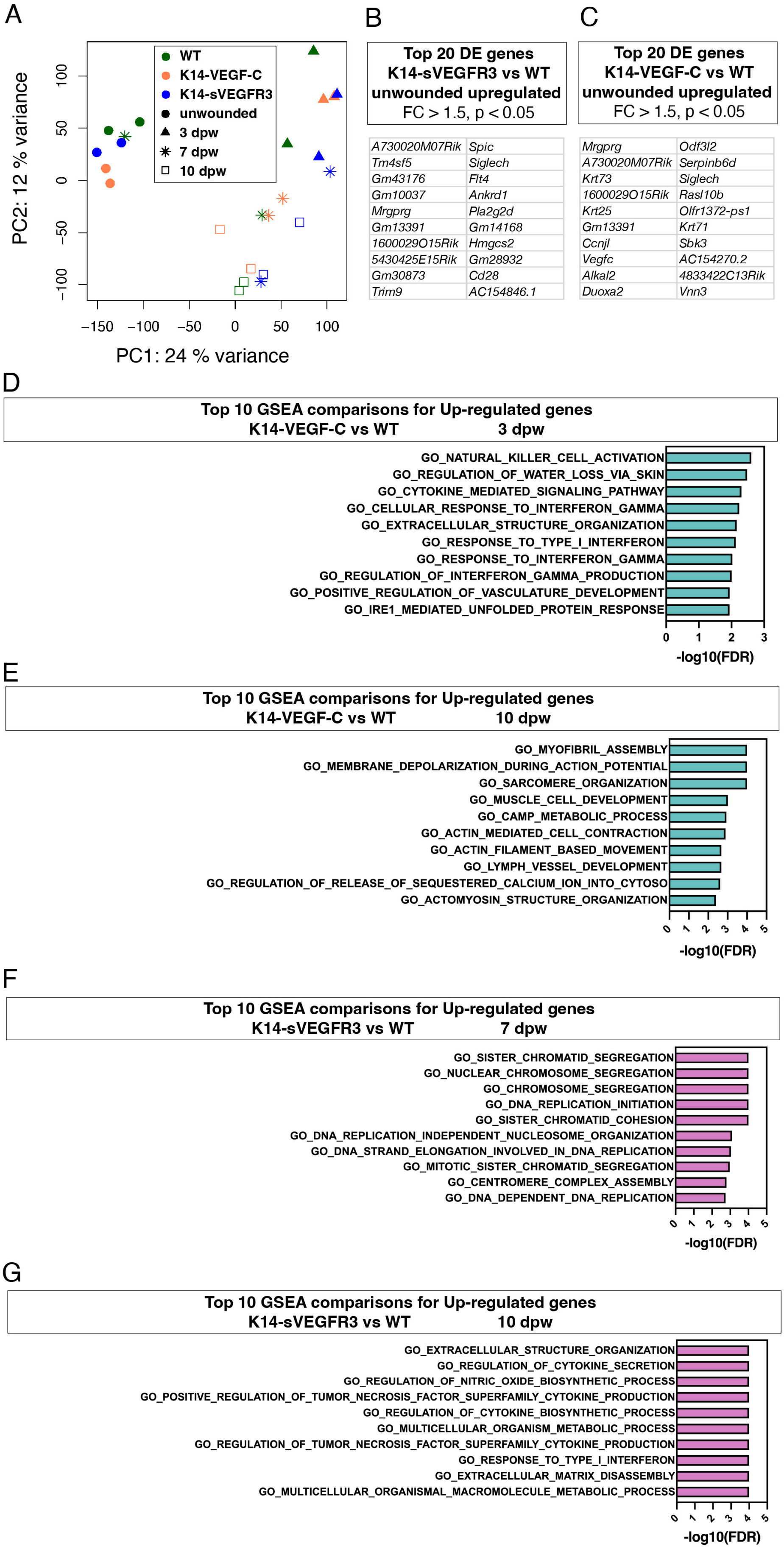

Figure S2. Gene set enrichment analysis (GSEA) from whole-wound RNA bulk sequencing of wildtype (WT), K14-sVEGFR3 and K14-VEGF-C transgenic mice. (A) Principal component (PC) analysis. **(B)** Top 20 differentially expressed

(DE) genes in K14-sVEGFR3 transgenic mice versus WT mice in unwounded skin. **(C)** Top 20 DE genes in K14-VEGF-C transgenic mice versus WT mice in unwounded skin. **(D)** Top 10 GSEA comparisons for upregulated genes in K14-VEGF-C transgenic mice versus WT mice at 3 days post-wounding (dpw). **(E)** Top 10 GSEA comparisons for upregulated genes in K14-VEGF-C transgenic mice versus WT mice at 10 dpw. **(F)** Top 10 GSEA comparisons for upregulated genes in K14-sVEGFR3 transgenic mice versus WT mice at 7 dpw. **(G)** Top 10 GSEA comparisons for upregulated genes in K14-sVEGFR3 transgenic mice versus WT mice at 10 dpw. n = 2 wounds per condition.

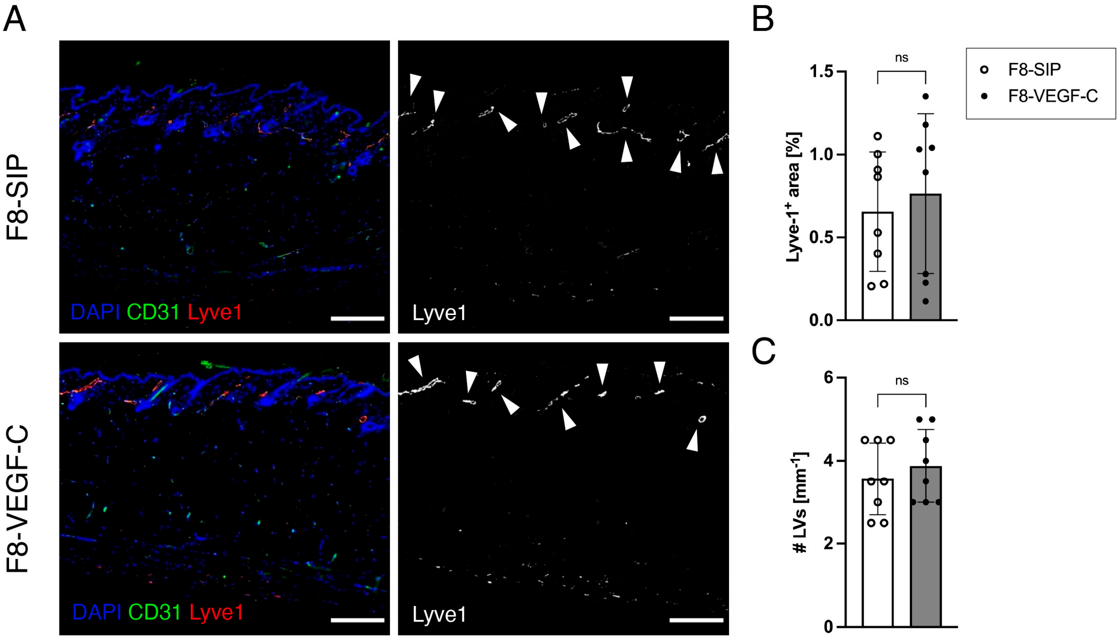

Figure S3. Treatment with F8-SIP and F8-VEGF-C has no effect on LVs in unwounded diabetic skin. (A) LYVE1-CD31 representative sections of diabetic unwounded back skin. Right: Single-channel image of Lyve1$^+$ staining. Scale bars: 500 µm. White arrowheads depict LVs. **(B)** Quantification of the LYVE1$^+$ area [%] of LVs in representative regions of unwounded sections. **(C)** Quantification of the number of LVs [mm^{-1}] in representative regions of unwounded sections. N = 8 wounds.

A

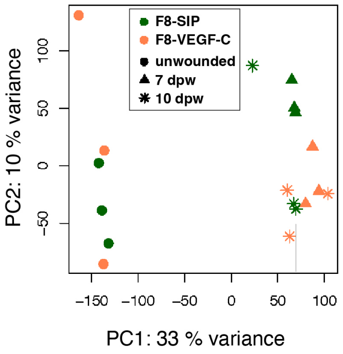

B

**Top 50 DE genes F8-VEGF-C vs F8-SIP 7 dpw** FC > 1.5, $p < 0.05$ up	down
Ms4a3	Chrdl2
Igkv1-135	Gm32352
Gm14085	2310001K20Rik
Fam135b	Gm29015
Gm17641	Gm21013
Igkv15-103	4932443L11Rik
4930461G14Rik	Odf3l2
Lyzl4	AC122418.1
Gm23711	Platr29
Camp	Krt25
Pax2	AC161812.1
Gm5045	AC140426.1
2010308F09Rik	Spink4
Vwa5b1	Gm47845
C230099D08Rik	Gm3671
Pax5	Mrgprb3
Gm12993	Gm37014
Gm24289	Gm11934
Gm43176	Prss23os
Adh4	Gm42648
Gm21388	AF529169
Fcer2a	Lrrc66
Gm11626	Pkd2l1
Cyp24a1	Mhrt
Gm15016	1700039I01Rik
Gm45643	Gm13450
Gm4258	4930570N18Rik
Gm13201	Rbm6-ps2
4930592C13Rik	Myhas
Gm5364	B930018H19Rik
Gm27048	Gm42858
Igkv8-30	AC158554.1
Gm48495	Gm37043
Gm45336	Kcnh7
AC159006.2	Kng1
Gm44929	Olfr266
Spata31d1b	Usp44
Gm28455	Skor1
4933406J09Rik	AL589670.1
Gm9182	Gm40038
Cr2	Gm4953
Slc16a5	Gm45778
Crnn	Gm43726
Ffar1	Gm48690
Gm38124	Gm30173
Ibsp	1700084C06Rik
Elane	Phactr3
Iglc3	Gm28610
Hapln1	AC154227.1
Cyp2d26	Gm15179

C

**Top 50 DE genes F8-VEGF-C vs F8-SIP 10 dpw** FC > 1.5, $p < 0.05$ up	down
Slc26a4	Fam71a
Krt33a	Gm6562
Gm6286	BC106175
Krtap7-1	Agmat
Ighv1-15	Rpl9-ps1
Gm29187	Igkv6-13
Gm17660	Ostn
CT009518.1	Cbln1
Ighv3-8	Gm26887
Krt71	1700016C15Rik
Hbq1b	Gm15340
Gm11574	Gm18492
Gm48660	Ifnab
Ceacam10	Gm11243
Krt25	Gm13073
Gm6341	Cyp2c52-ps
Ighv5-17	Ighv1-80
Psg22	Prl8a1
Serpinb3c	Apoa1
Krt87	Gm12596
Gm10330	Myl10
4930471M09Rik	Ppp1r1c
Ak9	Actl7b
Ighg3	Gm10913
4732487G21Rik	Olfr373
Gm44697	Gm48761
Il22	Igkv14-100
Gm15697	A730036I17Rik
Gm45822	Gm5581
Gm47148	Cc2d2b
Gm20468	Olfr374
Gm29459	Gm5050
Igkv5-39	Gm22656
Lce3a	Psg18
Klrb1-ps1	Psg28
4933440M02Rik	Gm13450
Gm16044	Gm27019
B930092H01Rik	Mup17
Hes2	Gm45301
Gm20392	Enthd1
Gm26831	Gm17473
Krt73	4931431B13Rik
AC103674.4	Gm24944
Prop1	BB014433
Cdk19os	Btnl1
Trim10	Gm9320
Gm47709	Csf3
Oit1	Gm43689
Sprr2f	Gm8319
Serpinb3d	Gm5100

D

**Top 20 GSEA comparisons for Up-regulated genes F8-VEGF-C vs F8-SIP 10 dpw** FDR < 0.05
Sister chromatid segregation
Nuclear chromosome segregation
Chromosome segregation
Sister chromatid cohesion
Mitotic nuclear division
Mitotic sister chromatid segregation
Mitotic spindle organization
Metaphase plate congression
Mitotic cytokinesis
Chromosome localization
Cilium organization
Cilium morphogenesis
Smoothened signaling pathway
Cytoskeleton dependent cytokinesis
Dna recombination
Cell division
Centromere complex assembly
Organelle fission
Reciprocal dna recombination
Spindle assembly

Figure S4. Gene set enrichment analysis (GSEA) from whole-wound RNA bulk sequencing of F8-SIP and F8-VEGF-C treated diabetic mice. (A) Principal component (PC) analysis. **(B), (C)** Top 50 differentially expressed (DE) up- and downregulated genes F8-VEGF-C vs F8-SIP treated diabetic mice at 7 and 10 days post-wounding (dpw) respectively. **(D)** Top 20 GSEA comparisons for upregulated genes in F8-VEGF-C vs F8-SIP treated diabetic mice at 10 dpw respectively. n = 3 wounds per condition.

Table S1. Patient information for human chronic wound samples.

Age	Gender	Clinical information	Type of chronic wound	Location
59	m	Arterial hypertension	Martorell's ulcer hypertonicum	Fibula left
68	f	Pronounced lymphedema lower leg both sides, chronic renal failure stage III, coronary artery disease	Ulcus cruris	Lower leg right
-	-	-	Venous leg ulcer	Lower leg right
69	f	Diabetes mellitus type 2, arterial hypertension, obesity per magna, gonarthrosis, chronic venous insufficiency	Martorell’s ulcer hypertonicum	-
81	m	Chronic obstructive pulmonary disease, diabetes mellitus type 2, arterial hypertension	Martorell’s ulcer hypertonicum	Lower leg right
94	m	Diabetes mellitus type 2, arterial hypertension, chronic venous insufficiency	Martorell’s ulcer hypertonicum	-
75	m	Diabetes mellitus type 2, Klinefelter's disease	Venous ulcer	-
74	m	Arterial hypertension	Deep dissecting hematoma Malleolus	Lower leg left distolateral

m = male, f = female
